# Emphasis on Quality of Life in Children and Adolescents With Bronchial Asthma

**DOI:** 10.7759/cureus.68762

**Published:** 2024-09-06

**Authors:** Vineeta Pande, Mrinali Thakur

**Affiliations:** 1 Pediatrics, Dr. D.Y. Patil Medical College, Hospital and Research Centre, Dr. D. Y. Patil Vidyapeeth (Deemed to be University) Pimpri, Pune, IND

**Keywords:** adolescents, bronchial asthma, children, quality of life (qol), standard of living

## Abstract

Quality of life (QoL) encompasses the overall well-being of individuals or populations, addressing both positive and negative elements at specific points in time. It is critical to recognize that mere existence is insufficient; the standard of living plays a vital role. The domains of symptom, emotion and activity need to be focused and areas requiring intervention to enhance individual and societal health should be understood, thus alleviating burdens on society, government, and healthcare systems. Bronchial asthma is one such area.

Untreated asthma correlates with higher absenteeism and poorer academic performance in children and there is an overall impact on their overall well-being. Research shows that proper medical treatment and counseling significantly improve QoL in asthmatic children, particularly in the activity and symptom domains, though the emotional domain often shows no significant improvement.

In India, limited studies have assessed QoL domains in asthmatic children, and even fewer have implemented steps to improve QoL. Evaluating QoL helps assess treatment efficacy and the need for further care, underscoring the importance of addressing asthma’s long-term effects on children’s well-being.

## Editorial

Quality of life (QoL) is a concept that aims to capture the well-being, both positive and negative elements, whether of a population or individual, within the entirety of their existence at a specific point in time. In the fast-developing and moving world, we often forget that mere existence is not important but the standard of living is crucial as well. Quality of life is measured in three domains: the symptoms domain, the emotional domain and the activity domain [1]. These domains help us identify the different aspects of a person’s life that require attention to improvement the overall health of the individual and society and to eventually help in reducing the imposed burden on the society, government and health facilities. One such area that needs focus is bronchial asthma in children and adolescents. 

Bronchial asthma is a chronic inflammatory disorder of the respiratory tract that occurs due to various endogenous and exogenous factors such as weather change, cold, dust, dandruff, pets and exercise. These factors trigger inflammation in the lower respiratory tract which causes mucus production, airway remodeling and bronchial hyperresponsiveness. Acute exacerbations, in the form of wheezing, discomfort while breathing and coughing occur frequently if the affected individual doesn’t take proper treatment and precaution. The prevalence of asthma symptoms in Global Asthma Network (GAN) Phase I for children was 9.1% and for adolescents was 11.0% [2]. Indian studies have suggested an incidence ranging from 2-18.2% in Indian children [3,4]. Many studies have shown an increased prevalence of asthma due to rapid urbanization which causes an increase in air pollutants [5]. These pollutants trigger the respiratory tract and cause bronchoconstriction in sensitized individuals, which precipitates an acute attack. Environmental tobacco smoke (ETS) has been associated with an increased upper respiratory tract infection incidence, persistence of asthma, and higher severity of asthma exacerbations [6]. The key to improving the quality of life for children and adolescents with asthma lies in a multifaceted approach that goes beyond symptom control. It is required to give attention to both physical and emotional aspects of living with chronic illness.

First and foremost, education and counselling are crucial. Parents, children and even teachers need to understand asthma and its triggers. By empowering young people to take control of their condition through asthma action plans, there can be a reduction in the number of emergency room visits and school absenteeism. Children with untreated asthma have known to have higher rate of absenteeism. This causes the child to miss classes, decrease the number of school days and can cause an overall decline in the scholastic performance and school related activity. School absenteeism, in a study done by Hsu et al., was noted to be higher in children with partly controlled or very properly controlled bronchial asthma with a prevalence ratio of 1.5 (95% CI: 1.34-1.69) and even emergency hospital visits for asthma (prevalence ratio: 3.27; 95% CI: 2.44-4.38) [7].

When children understand their medications and know how to manage their symptoms, they can manage fully in everyday activities. The involvement of families is indispensable. They play a vital role in encouraging children to adhere to treatment plans, recognize early signs of exacerbations and seek help when necessary. Studies revealed that family empowerment done by educating the family members enhanced the QoL of children and parents, improved asthma control as well as improvement in pulmonary function and a reduction in parental stress [8-10] Many studies have also shown that more than half of the asthmatic subjects discontinue medications once their symptoms have been controlled [11]. This issue is also of grave importance as it leads to significant adverse effects on the child and leads to greater noncompliance with the treatment. 

Bronchial asthma can significantly impact the quality of life in children, affecting their physical, emotional, and social well-being. Some aspects that may be influenced are: symptoms and severity where there can be an increase in the frequency and intensity of asthma attack, wheezing, coughing and shortness of breath; emotional and mental health where anxiety, fear and stress related to exacerbations an cause a decrease in self-esteem and confidence, social interactions and relationships are affected as they find difficulty in participating in sports, play or social activities which can lead to isolation or exclusion; absenteeism, reduced focus and decreased energy levels can impact academic achievement and overall educational experience hence reducing their school and academic performance, family and caregiver burden, treatment and medication, lifestyle medication, stigma and self-perception, economic impact and comorbidities.

Studies have shown that there is moderate to severe impairment in QoL in paediatric age groups who weren’t given proper counseling or those who eventually were lost to follow up. But an improvement in the quality of life is seen when children receive proper access to medical treatment. A study done by Battula et al. [12], using the Paediatric Asthma Quality of Life Questionnaire (PAQLQ), concluded that there was a significant change in the total score quality of life in the post-treatment analysis and counseling after four weeks. 

Another questionnaire-based study by Wander et al. [13] showed statistically significant improvement in two domains, the activity limitation domain and the symptom domain of the mini-PAQLQ post-treatment. There was also a notable change in the mean total scores that showed an overall improvement in the condition of the child with medical intervention. However, there was no significant change in the emotional domain of the QoL questionnaire even after medical intervention.

In a study done by Savdahiya et al. [14], it was found that 100 asthmatic children showed improvement in all three domain and overall QoL after four weeks of standard treatment. But in children with severe asthma with activity limitation, there was no improvement in the emotional score. Most of the studies have shown to have a significant improvement in the activity and symptoms domain in follow-up studies which helps us understand the need and importance of counseling as well as the importance of evaluation of QoL. 

The immediate effect of poor quality of life is seen as increased absenteeism in school, decreased participation in school activities and indirectly affecting IQ of the child. In adolescents, it is noted that there is an increased chance of psychological and social impairment such as depression, anxiety and an increasing BMI which causes in increase in other co-morbidities [15]. Psychological interventions such as behaviour therapy, cognitive therapy relaxation techniques along with positive cognitive experience sharing regarding asthma have been shown to be associated with increase in adherence, decreased emotional outbursts, reduction in psychological distress and overall helps in better asthma control [16,17]. 

In India, there are only limited studies available that have assessed these domains and even fewer, where the steps to improve the quality of life have been taken. National-level interventions are needed to control asthma such as access to good quality healthcare, public awareness and education, environmental control, reduction in socioeconomic barriers, support systems such as psychological, parental and community support along with comprehensive asthma programs, school health policies and training and capacity building. Improving quality of life for children and adolescents with asthma is not just about managing symptoms; it's about enabling them to lead healthy, active, productive fulfilling lives. Asthma is more than just a medical condition, rather a factor that influences every aspect of a child's life. Hence, it is essential for the community and the healthcare system to work together to provide a holistic approach to help children and adolescents live a healthy life and thrive.
